# Scientific Items

**Published:** 1888-06

**Authors:** 


### SCIENTIFIC ITEMS.

**The time in which We Think.**—One of the most beautiful applications of electricity which has of late been made is its use in the study of pyschological phenomena And why, indeed, is not the subtle power by which time and space are being annihilated, and human labor rendered less irksome, the most proper agent to assist man in the study of the facts of his own conciousness? In an elaborate article in the *Nineteenth Century*, Dr. J. McK. Cattell gives an account of the time measurements of thought made by means of the line drawn on a rapidly moving surface by a pen attached to the prong of a tuning fork vibrating at a constant rate, by means of electricity. By a delicate aparatus constructed on this principle, duration of time may be measured to the one ten-thousandth of a second. The writer above named has found that the process varies in its degree of rapidity in different individuals, children and old persons thinking slower than people of middle age, ignorant persons thinking more slowly than educated persons. In this way he also found he could measure the time it take to perceive, that is, the time which passes from the moment when the impression reaches conciousness until the moment at which we know what it is. In his own case he found that it took 1-20 second to see white light, 1-10 second to see a picture, 1-8 to see a letter, and 1 7 to see a word. It takes longer to see a rare word than a common word, or a word in a foreign language than in our native tongue. It even takes longer to see some letters than others. "Will time," or time taken up in choosing, can be measured. It takes 1-13 second to judge between blue and red. To recall the name of a printed word takes 1-9 second, to a letter 1-6 second, to a picture 1-4 second. It takes less time to remember the name of a familliar word than of a letter, though it takes less time to see the letter. The time of remembering can be measured. It takes 1-4 second to translate a word from one language to an ther when you are familiar with both. It takes 1 20 second longer to translate a word from a foreign lauguage to your native tongue than it does in the other direction. We can think of the name of the next month in half the time we can think of the last month. It has been demonstrated that sensation does not travel through the nerves to the brain so fast as has been supposed. Its speed is not much greater than sixty miles and hour.—*Light and Heat.*

**The Origin of Petroleum.**—Professor Medelejef has advanced the theory that petroleum is of mineral origin, and that its production is going on and may continue almost indefinitely. He has succeeded in making it artificially by a similar process to heat which he believes is going on in the earth; and experts find it impossible to distinguish between the natural and the manufactured article. His hypothesis is that water finds its way below the crust of the earth and then meets with carbonides of metals (particularly of iron) in a glowing state. The water is decomposed into its constituent gases. The oxygen unites with the iron, while the hydrogen takes up the carbon and ascends to a higher region, where part of it is condensed into mineral oil, and part remains as natural gas, to escape where it can find and outlet, or to remain stored at great pressure until a borehole is put down to provide it a passage to the surface. Oil-baring strata occur in the vicinity of mountain ranges; and it is supposed that the upheaval of the hills has sufficiently dislocated the strata below to give the water access to depths from which it is ordinarily shut out.—*Scientific American.*

**A New Flameless Explosive.**—A new variety of "securite" has heen prepared by Herr Schoenweg, which is said to be flameless when exploded, and will, it is expected, be of especial value as a substitute for ordinary blasting powder and other explosives in fiery coal mines. It consists of nitrated hydrocarbons mixed with an oxodizing agent, such as chlorate of potash and some organic salt which renders the mixture flameless. The substance is not hydroscopic, and is of a bright yellow color, and can be kept for any length of time without undergoing any change. It cannot be exploded by a flame nor by a hot substance, but only by a detonating cap. Recent experiments at Hendon have proved that the new explosive fulfills the anticipations of the inventor, and we understand that the Flameless Explosives Company have undertaken to introduce it to the notice of mine owners and others to whom an explosive of this nature should be welcome. Its power is said to be equal to that of No 1 dynamite, and it can be manufactured at a less cost. The organic salt which is added to the "securite" to produce this effect has also the property of rendering dynamite similarly flameless when mixed with it.—*Scientific American.*

**Origin of Meteorites**—From an exhaustive study of the very large collection of meteorites at Harvard College, the conclusion has been arrived at that many of the masses of meteoric iron now known are cleavage crystals, broken off probably by the impact of the masses show cleavings parallel to the planes of all the three fundamental forms of the isometric or regular system; the Widmanstatten figures and Neumann lines are sections of planes of crystalline growth parallel to the same three fundamental forms of the isometric system, and, on different sections of meteorites, Widmanstatten figures and Neumann lines can be exhibited in every degree, with no break where a natural line of division can be drawn. The features of the Widmanstatten figures are due to the elimination of incompatible material during the process of crystallization, and the results of this investigation confirm the theory that the process of crystallization must be very slow. From all that appears, the theory has come to be entertained, in respect to the origin of meteorites, that the masses were thrown off from a sun among the fixed stars, and that they were slowly cooled while revolving in a zone of intense heat.—*Phrenological Journal.*

